# Validation of Soft Multipin Dry EEG Electrodes

**DOI:** 10.3390/s21206827

**Published:** 2021-10-14

**Authors:** Janne J.A. Heijs, Ruben Jan Havelaar, Patrique Fiedler, Richard J.A. van Wezel, Tjitske Heida

**Affiliations:** 1 TechMed Centre, Department of Biomedical Signals and Systems, University of Twente, 7522 NB Enschede, The Netherlands; r.j.a.vanwezel@utwente.nl or t.heida@utwente.nl (T.H.); 2 Donders Centre for Neuroscience, Department of Biophysics, Radboud University, 6525 AJ Nijmegen, The Netherlands; jurjan.havelaar2@donders.ru.nl; 3 Institute of Biomedical Engineering and Informatics, Technische Universität Ilmenau, 98693 Ilmenau, Germany; patrique.fiedler@tu-ilmenau.de

**Keywords:** electroencephalography (EEG), dry electrodes, gel electrodes, brain imaging, validation study

## Abstract

Current developments towards multipin, dry electrodes in electroencephalography (EEG) are promising for applications in non-laboratory environments. Dry electrodes do not require the application of conductive gel, which mostly confines the use of gel EEG systems to the laboratory environment. The aim of this study is to validate soft, multipin, dry EEG electrodes by comparing their performance to conventional gel EEG electrodes. Fifteen healthy volunteers performed three tasks, with a 32-channel gel EEG system and a 32-channel dry EEG system: the 40 Hz Auditory Steady-State Response (ASSR), the checkerboard paradigm, and an eyes open/closed task. Within-subject analyses were performed to compare the signal quality in the time, frequency, and spatial domains. The results showed strong similarities between the two systems in the time and frequency domains, with strong correlations of the visual (ρ = 0.89) and auditory evoked potential (ρ = 0.81), and moderate to strong correlations for the alpha band during eye closure (ρ = 0.81–0.86) and the 40 Hz-ASSR power (ρ = 0.66–0.72), respectively. However, delta and theta band power was significantly increased, and the signal-to-noise ratio was significantly decreased for the dry EEG system. Topographical distributions were comparable for both systems. Moreover, the application time of the dry EEG system was significantly shorter (8 min). It can be concluded that the soft, multipin dry EEG system can be used in brain activity research with similar accuracy as conventional gel electrodes.

## 1. Introduction

Electroencephalography (EEG) is an electrophysiological measurement technique that records the electrical activity of the cerebral cortex with a high temporal resolution, using non-invasive scalp electrodes [1]. In research, it is a widely used technique to investigate visual, auditory, and cognitive functioning, as well as to gain insights, for instance, into the cortical pathways and impairments involved in neurological (movement) disorders [2], such as Parkinson’s disease [3] or stroke [4]. More recently, EEG has become an important tool in research on brain–computer interfaces [5,6]. In addition to its utility in research, EEG is used clinically as a diagnostic tool for diseases such as epilepsy or sleep disorders [7,8]. It is likely that the applicability of EEG systems will be extended in the future because of current developments towards mobile EEG systems and developments in the design of the electrodes.

The gold standard in EEG sensors is the use of conventional gel electrodes. These electrodes require the application of conductive gel, which serves as a conductive medium to bridge the gap between the subject’s head and the electrodes, thereby reducing the electrode-skin impedance [1]. Gel EEG systems are known for their high signal quality and their good comfort. However, as frequently reported, the need for conductive gel yields some disadvantages [9]. First, expertise and experience are needed for the application of the conductive gel. Second, the application of gel can be time-consuming [10,11,12,13,14]. Third, measurement time is limited because of the drying of the gel, which decreases the electrode-skin impedance. For instance, after a measurement time of 5 h, a reduction in electrode-skin impedance of 10 kΩ was reported [15]. Lastly, cleaning the subject’s hair and the EEG cap after the experiment takes additional time. These disadvantages restrict the use of conventional gel EEG systems to mainly the laboratory environment.

Development of EEG systems using gel-free, i.e., dry, sensors, show promising results to overcome these problems and will enable the recording of brain activity outside the laboratory [9,16]. The most common concept in dry-contact electrodes is the multipin shape [9,11]. Multipin electrodes consist of multiple pins that pass through the hair layer to establish direct physical contact with the scalp. Therefore, these dry-contact multipin electrodes do not require the application of conductive gel.

The fact that dry electrodes do not require the application of gel results in some advantages and disadvantages. First, the preparation and application of the dry EEG system are simplified. Some studies even reported that the EEG cap could be applied by the subjects themselves [14]. Second, the application time is reduced in comparison to gel EEG systems. Previous studies found a reduction in application time of 30% up to 89% for the dry EEG system in comparison to a conventional gel EEG system [10,11,12,14]. However, the omission of conductive gel comes at the expense of high electrode-skin impedances. As a consequence, higher channel rejection rates [12,13,14] and reduced signal-to-noise ratios were reported for dry electrodes [17,18]. Therefore, it is important to compare the signal quality of dry EEG electrodes with the quality of conventional gel EEG electrodes to determine whether the characteristic brain responses in the time, frequency, and spatial domains can be measured similarly by both systems. However, literature can be ambiguous on the performance of dry EEG systems because of the high variability in the design of dry electrodes and the different methodologies and tasks used to validate systems [19].

Previous studies reported high similarities between measurements using dry and gel EEG electrodes in both the frequency and time domains. In the frequency domain, high correlations in power were observed [10,12,13,14,17,18,20], especially for the alpha band (8–14 Hz) during an eyes open/closed task [13,18]. However, increased power in the lower delta frequency band (1–4 Hz) [11,12,13,14,18,20] (or even in all frequency bands [17]) has frequently been reported for dry electrodes, possibly due to sweating or mechanical deformation of the skin. In the time domain, the signals of dry and gel electrodes are highly correlated, as evaluated with event-related potentials (ERP) [11,13,14]. However, slightly increased noise levels were observed for dry electrodes [14,17]. Moreover, high similarities in the spatial domain were reported, with only 0.5% up to 3.5% of the pixels that were significantly different between the two systems [11].

Another important consideration in the evaluation of dry electrodes is the comfort experienced by the subject. Gel EEG systems are known for their good comfort, while the comfort of some dry EEG systems needs improvement [10,13,19], especially for the frontal-temporal regions [12]. The pressure of the pins of dry electrodes on the head reduces comfort, specifically when single-pin dry electrodes are used [10]. Fiedler et al. evaluated the design of dry electrodes to improve both signal quality and comfort [21,22]. First, they evaluated the size of the electrodes and the number of pins per electrode. Smaller electrodes can pass more easily through the hair layer. However, by reducing the number of pins, the local pressure force on the head increases, reducing the comfort of electrodes. Therefore, a compromise was made between the number of pins affecting signal quality and the contact force affecting the comfort of the electrodes. Second, the length of the pins was made variable, with longer pins for head regions with dense, long hair and small pin lengths for regions with short or no hair. This allowed the pins to pass through the hair layer while preserving comfort. Lastly, the stiffness of the pins was evaluated to seek a balance between signal quality and comfort. The stiffer the material of the electrode, the easier it passes through the hair layer to maintain a constant physical connection to the scalp, and the less susceptible the electrodes are for motion artifacts; however, the more flexible the material, the better the comfort of the electrodes.

Since the performance of the soft, dry EEG system was evaluated on a bipolar electrode setup only [21,22], the aim of the current study is to evaluate the performance and signal quality of soft, dry EEG electrodes in comparison to conventional gel electrodes in a realistic multichannel array. The general performance (i.e., application time, experienced comfort, and the percentage of rejected data) and signal quality in the time, frequency, and spatial domains, are evaluated by means of three tasks.

- The checkerboard paradigm (CHECK) is used to compare systems in the time and spatial domains. The checkerboard paradigm evokes the pattern-reversal visual evoked potential (VEP). This VEP is known for its low variability within and between subjects [23,24] and can be used to evaluate visual functioning in a variety of diseases (reviewed in [23]).
- The 40 Hz auditory steady-state response (ASSR)-task is used to compare systems in the time, frequency, and spatial domains. The ASSR is an auditory evoked potential (AEP), which can be used to evaluate hearing thresholds [25,26], and is a potential biomarker for psychiatric disorders such as schizophrenia [27]. Amplitude modulated tones evoke a synchronous activity in the auditory pathways, which can be observed as a large peak at the modulation frequency in the power spectral density (PSD) [25,28].
- The eyes open/eyes closed (EOEC)-task is used to compare systems in the frequency and spatial domains. The EOEC-task is frequently used in studies that validate newly developed EEG electrodes [10,11,12,13,14,18]. When closing the eyes, an increase in alpha-band activity can be observed (i.e., Berger effect [29]), which then reduces immediately when opening the eyes.

Compared to previous studies using multipin electrodes, it is hypothesized that application time will be reduced, and experienced comfort will be improved for the soft multipin dry EEG system. Moreover, it is expected that similar patterns will be observed for EEG analyzed in the time, frequency, and spatial domains, with slightly increased noise levels for the soft, multipin dry EEG electrodes compared to the gel electrodes.

## 2. Materials and Methods

### 2.1. Participants

Fifteen healthy subjects (8 males, 7 females) participated in the study. They had an average age of 22.7 ± 2.2 years (mean ± SD), and an average head circumference of 56.3 ± 1.9 cm (mean ± SD). Subjects were included if they were between 18 and 30 years old. They were excluded from participation if they suffered from a skin disease affecting the skin on the head, used drugs affecting the central nervous system (e.g., antidepressant, antipsychotics), had brain surgery in the past, suffered from epilepsy, suffered from a psychiatric or neurological disease in the past year, or if they had a history of substance abuse in the past year. All subjects provided written informed consent prior to the experiment.

### 2.2. Data Acquisition

The gel EEG system (waveguard original; ANT Neuro b.v., Hengelo, The Netherlands) consisted of 32 silver/silver-chloride (Ag/AgCl) electrodes positioned according to the 10-10 international system. Two integrated electrodes at AFz and CPz were used as the ground and reference, respectively. Electrode-skin impedance levels of the electrodes were aimed to be below 20 kΩ by using conductive gel. The average impedance level of the gel electrodes (with the exception of the ground and reference) was 6.8 ± 5.6 kΩ (mean ± SD; standard deviation).

**Figure 1:**
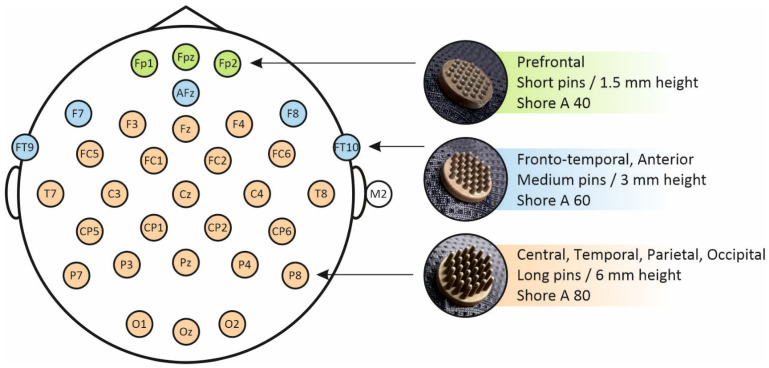
The 32-channel layout of the soft, multipin, dry EEG system, indicating the location and shore hardness of short pins (green), medium pins (blue), and long pins (orange).

The dry EEG system (waveguard touch; ANT Neuro b.v., Hengelo, The Netherlands) consisted of 32 soft, multipin electrodes positioned according to the 10-10 international system. The electrodes were made of a polyurethane (PU) substrate. All electrodes were coated using a silver-chloride (AgCl) electroless plating technique [11]. Electrodes had a diameter of 1.5 cm and comprised 30 pins. The length of the pins was variable in accordance with the head region (Figure 1). Therefore, short pin lengths (1.5 mm) were used for the prefrontal area, medium pin lengths (3 mm) for the frontal-temporal and anterior areas, and long pin lengths (6 mm) for the frontal-central, central, temporal, parietal, and occipital areas. In contrast to previous studies using electrodes with a homogeneous shore hardness of A98 [11,12], the present study used electrodes with flexibility selected specifically for each electrode pin length and head region (Figure 1). The electrodes with short pins comprised a substrate of Shore A 40. Electrodes with medium pins were based on substrates of Shore A 60. The electrodes comprising long pins had a shore hardness of A 80. Two external self-adhesive hydrogel AgCl disc electrodes at the left and right mastoid were used as ground and reference, respectively. The impedance of the ground and reference were aimed to be below 20 kΩ by cleaning the skin with alcohol and using a tiny drop of conductive gel on the ring electrodes. A specialized dry EEG software module (eego^TM^ software v1.9.1; ANT Neuro b.v., Hengelo, The Netherlands) was used to observe and improve the signal quality of the dry electrodes. This module assesses the signal quality of each electrode separately by a peak-to-peak amplitude threshold of 400 μV in a 1 s sliding window. A channel reliability of at least 80% was aimed for. The average impedance level of the dry electrodes (with the exception of the ground and reference) was 601.5 ± 400.8 kΩ (mean ± SD).

For both EEG systems, data was collected using the eego^TM^ amplifier (EE-225; ANT Neuro b.v., Hengelo, The Netherlands), with a sampling frequency of 1024 Hz.

### 2.3. Experimental Protocol

All subjects performed two experimental sessions at the Radboud University, Nijmegen, in a room with dimmed lights. The two sessions occurred on separate days but at the same part of the day. During the sessions, either the dry EEG system or the gel EEG system was tested in a pseudorandomized order between subjects. In both sessions, subjects performed three tasks: the checkerboard paradigm, the auditory steady-state response, and the eyes-open/eyes-closed task. The order of the tasks was similar for both experimental sessions within a subject but randomized between subjects. The MATLAB toolbox, Psychtoolbox-3 [30], was used for the presentation of visual stimuli and sound stimuli via a standard laptop. Sound stimuli and instructions were presented via insert earphones (Etymotic ER1). Volume levels and display settings were set equally between sessions and subjects.

#### 2.3.1. Checkerboard Paradigm (CHECK)

During the checkerboard paradigm, the subject was asked to focus on a red dot in the middle of the screen. A 24-by-18 checkerboard pattern was presented with black and white checks (visual angle of 1°, distance to screen 65 cm). Every 0.45 s, the checkerboard reversed (i.e., black checks became white and vice versa). After a 10 s baseline, one block of 300 trials (i.e., checkerboard reversals) was obtained per subject. The pattern-reversal VEP, as evoked by the checkerboard paradigm, was used to compare both systems in the time and spatial domains.

#### 2.3.2. Auditory Steady-State Response (ASSR)

During the ASSR-task, 12 s audio segments were presented, with a jittered intertrial interval of 2 to 3 s. The subject was asked to focus on a red fixation point on the wall. The audio segments consisted of tones with a 500 Hz carrier frequency and a 40 Hz modulation frequency. In total, 75 trials of 12 s were obtained per subject, unequally divided over 3 blocks. Each block started with a 10 s baseline. The auditory evoked potential (AEP), occurring in the first second of each 12 s trial, was used to compare both systems in the time and spatial domains. Furthermore, the 40 Hz power (caused by the modulation component of the audio stimulus) was used to compare both systems in the frequency domain.

#### 2.3.3. Eyes Open/Eyes Closed Task (EOEC)

During the EOEC-task, the subjects alternately opened their eyes for 5 s when hearing the Dutch command ‘open’ (i.e., ‘open’) and closed their eyes for 3 s when hearing the Dutch command ‘dicht’ (i.e., ‘close’). When having their eyes opened, subjects were asked to focus on the wall in front of them without moving their head. In total, 60 trials (open and closed eyes) were obtained per subject, divided equally over 2 blocks. Each block started with a 10 s baseline. The power of the delta [1–4 Hz], theta [4–8 Hz], alpha [8–14 Hz], beta [14–30 Hz], and gamma [30–48 Hz] band were used to compare both systems in the frequency domain. Moreover, the topographical distribution of the alpha band was used to compare both systems in the spatial domain.

#### 2.3.4. Questionnaires

Before and after performing the tasks, subjects completed two short questionnaires. First, subjects indicated their level of sleepiness by the Karolinska Sleepiness Scale (KSS; 1 = extremely alert; 10 = extremely sleepy, cannot keep awake [31,32]). Second, they indicated their experienced comfort of the EEG cap with a 10-point Likert scale (1 = extremely comfortable; 10 = maximum imaginable pain).

### 2.4. Data Processing and Analysis

Pre-processing and analysis of the data were performed in MATLAB using eeglab (an open-source toolbox [33]). Electrodes that were not contained in both systems were removed from further analysis. Therefore, M1 and POz were removed for the gel EEG system, and AFz, FT9, and FT10 were removed for the dry EEG system. The 29 overlapping electrodes remained for further analyses. The data was filtered using a zero-phase, 2nd-order Butterworth bandpass filter (cut-off frequencies 1–48 Hz) and a zero-phase 2nd-order Butterworth bandstop filter (cut-off frequencies 48–52 Hz) to remove 50 Hz line noise. Bad channels with an average amplitude below 1 μV (flat line) or above 50 μV were removed from further analyses. The independent component analysis was applied to remove eye blink artifacts. The eye blink component was selected based on the following properties [34]: (1) the topographical distribution of the independent component shows activity in the frontal electrodes only, (2) the characteristic pattern of eye blinks can be observed in the independent component over time, and (3) the eyeblink component is present over the entire course of the experiment and is not linked to a specific task. Moreover, the reference was set equal for both EEG systems. Therefore, the data of the gel EEG system was re-referenced to the right mastoid (M2), similarly as for the dry EEG system. Subsequently, the data of each task was extracted, including an additional 5 s before and after the start and end of the task, respectively. For each of the tasks separately, bad channels were removed based on three criteria: (1) if the channel’s average amplitude within the specific task was below 1 μV or above 50 μV, (2) if the channel’s average amplitude within the specific task exceeded the mean + 2xSD over all channels within the task, or (3) if the channel’s average power within the specific task exceeded the mean + 2xSD over all channels within the task in at least 2 out of 5 frequency bands (delta [1–4 Hz], theta [4–8 Hz], alpha [8–14 Hz], beta [14–30 Hz], gamma [30–48 Hz]). For the third criterium, the average power over the task-specific trials was calculated using a Hann window (window length: VEP 0.5 s; AEP 0.8 s; 40 Hz-ASSR 11 s; EOEC 3 s).

#### 2.4.1. Performance in the Time Domain

The VEP evoked by the checkerboard paradigm and the AEP evoked by the ASSR-task were used to compare the EEG systems in the time domain. Additional pre-processing steps were applied. First, the data were filtered using a zero-phase, 2nd-order Butterworth bandpass filter (cut-off frequencies 1–30 Hz). Second, data were divided into trials of 500 ms for the VEP and 800 ms for the AEP, including a pre-stimulus baseline of −100 ms. Baseline correction was performed by subtracting the channel’s average baseline per trial. Lastly, bad trials were removed per channel: (1) if the channel’s average amplitude exceeded the threshold (mean + 2xSD over all trials) during the specific trial, or (2) if the channel’s average power exceeded the threshold (mean + 2xSD over all trials) in at least 2 out of 4 frequency bands (delta, theta, alpha, beta) during the specific trial.

After pre-processing, the VEP and AEP were averaged over all remaining trials. Moreover, the global field power in the time domain (GFPt) was calculated (Equation (1); [11,35]).

(1) $$\mathrm{GFPt}=\sqrt{\frac{1}{2n}\;\sum_{i=1}^{n}\sum_{j=1}^{n}{\left(U_{i}-U_{j}\right)}^{2}}$$

The GFPt is a measure of spatial standard deviation over all electrodes *n* at a certain point in time *t*. It takes into account the voltage difference *U* between all pairs of electrodes *i* and *j*. Thus, local activity at a certain time point can be observed by a high GFPt, while broadly spread activity is indicated by lower GFPt values.

To evaluate the similarities in GFPt between the gel and dry EEG system, the Spearman’s rank correlation (COR) and the root mean square deviation (RMSD) of the GFPt were calculated per subject [11].

#### 2.4.2. Performance in the Frequency Domain

Besides the AEP, the ASSR-task was used for comparison in the frequency domain by evaluating the 40 Hz peak (caused by the modulation frequency) in the power spectral density (PSD). Some additional pre-processing steps were taken. First, electrodes with a less pronounced or absent 40 Hz-ASSR response were removed. Therefore, T7, T8, P7, P8, O1, O2, and Oz were removed for both caps for all subjects [36]. Second, data were filtered using a zero-phase, 2nd-order Butterworth bandpass filter (cut-off frequencies 30–48 Hz). Third, the first 1 s of all trials was removed, eliminating the AEP. Last, using a nonoverlapping, sliding window of 1 s, bad windows were removed from trials: (1) if the channel’s average amplitude exceeded the threshold (mean + 2xSD over all trials) during the specific trial, or (2) if the channel’s average power in the gamma band exceeded the threshold (mean + 2xSD over all trials) during the specific trial.

After pre-processing, the PSD was calculated using a 4 s sliding Hann window with 75% overlap to obtain the power between 35 and 45 Hz with a frequency resolution of 0.5 Hz. Moreover, the PSD was standardized by calculating the Z-score. For both the absolute and standardized PSD, the average 40 Hz power and the signal-to-noise ratio (SNR) were obtained. The SNR (Equation (2)) was calculated by dividing the 40 Hz power *P* (i.e., signal) by the standard deviation of the power in the frequency window 35–39 Hz and 41–45 Hz (i.e., noise).

(2) $$\mathrm{SNR}=\frac{P{\;}_{\left[40\mathrm{Hz}\right]}}{\mathrm{std}\left(\;P_{\;\left[35-39\mathrm{Hz},41-45\mathrm{Hz}\right]}\;\right)}$$

In addition to the ASSR-task, the EOEC-task was also used for comparison in the frequency domain. Additional pre-processing steps were applied. First, eyes open (EO) and eyes closed (EC) trials were extracted separately. Since the duration of EC trials (3 s) was shorter than the duration of EO trials (5 s), the middle 3 s were taken for the latter condition. Second, using a nonoverlapping sliding window of 1 s, bad windows were removed from trials: (1) if the channel’s average amplitude exceeded the threshold (mean + 2xSD over all windows), or (2) if the channel’s average power exceeded the threshold (mean + 2xSD over all windows) in at least 2 out of 5 frequency bands (delta, theta, alpha, beta, gamma).

After the additional pre-processing steps, the PSD was calculated using a 1 s sliding Hann window with 50% overlap. The PSD was standardized by calculating the Z-score. The average power per frequency band was calculated for both the absolute and standardized PSD.

#### 2.4.3. Performance in the Spatial Domain

The standardized topographic distribution (Z-score) of the two main components of the VEP and AEP were generated for both systems. Therefore, the peaks of the average GFPt were used as time points to calculate the topographical distribution of the main components of the VEP (i.e., N75 and P100) and the AEP (i.e., N1 and P1). Moreover, the standardized topographic distribution of the average alpha band power (i.e., frequency of main interest in the EOEC-task) was calculated for both systems. Biharmonic spline interpolation was used for the areas in between electrodes.

#### 2.4.4. General Performance

To assess the general performance of both systems, the time needed for the application and preparation of the EEG systems was assessed (i.e., application time). The percentage of data that had been rejected in the pre-processing phase (i.e., bad channel rejection and bad trial/window rejection) was evaluated per channel for both systems. Moreover, the subject indicated the comfort of the cap on a 10-point Likert scale as described previously.

### 2.5. Statistical Analysis

The assumption of a normal distribution of the dependent variable failed, as assessed using the one-sample Kolmogorov–Smirnov test. Therefore, non-parametric statistical tests were performed with a significance level of α = 0.05. For statistical comparison between systems in the time domain, the Wilcoxon signed-rank test was used to examine differences in the COR and RMSD values of the VEP and AEP. For statistical comparison in the frequency domain, the Wilcoxon signed-rank test was used for both the absolute and standardized PSD to evaluate the subject’s averages over channels of the 40 Hz-ASSR power, the SNR, and the power per frequency band (EOEC-task). A Bonferroni correction was applied for the EOEC-task to adjust for the number of frequency bands (α = (0.05/5) = 0.01). Moreover, a within-subject correlation between all pairs of electrodes was performed for the 40 Hz-ASSR power, the SNR, and the power per frequency band, using Spearman’s rank correlation. For statistical comparison of the general performance, the Wilcoxon signed-rank test was performed to examine differences in the application time and the percentage of rejected data between the two systems. In addition, the non-parametric two-way repeated-measures ANOVA (i.e., Friedman’s test) was performed to evaluate differences in experienced comfort and sleepiness before and after the experiment.

The topographic distribution (spatial domain) of the main components of the VEP and AEP, and the distribution of the average alpha band power during the EOEC-task, were statistically compared using non-parametric permutation testing, including a pixel-based correction for multiple comparisons [37,38]. Therefore, a permutation distribution (*H*_0_) was created for each of the 3409 pixels in 23,768 iterations (2*^n^*, with *n* = 15 subjects). For each iteration, the condition label (i.e., ‘dry EEG’ or ‘gel EEG’) was permuted, and the t-statistic of the paired *t*-test was calculated (Equation (3)):

(3) $$t=\frac{\bar{X_{D}}}{S_{D}/\;\sqrt{n}}$$

with *X_D_* and *S_D_* as the average and standard deviation of the difference between the pairs and *n* as the number of pairs (*n* = 15 subjects).

Moreover, the minimum and maximum *t*-value over all pixels were saved for each iteration. Pixel-based correction for multiple comparisons was performed by calculating the statistical thresholds, defined as the 2.5th percentile of the smallest *t*-values (lower threshold) and the 97.5th percentile of the largest *t*-values (upper threshold). The observed test statistic (*obs*) for every pixel, using the real condition labels, was converted to a *Z*-value (Equation (4); [37]):

(4) $$Z=\frac{obs-\bar{H_{0}}}{S_{H_{0}}}$$

by subtracting the average value of the *H*_0_ permutation distribution and dividing the result by the standard deviation of the *H*_0_ permutation distribution. A pixel was significantly different between systems if its *Z*-value was smaller than the lower threshold or higher than the upper threshold.

## 3. Results

### 3.1. Performance in the Time Domain

**Figure 2:**
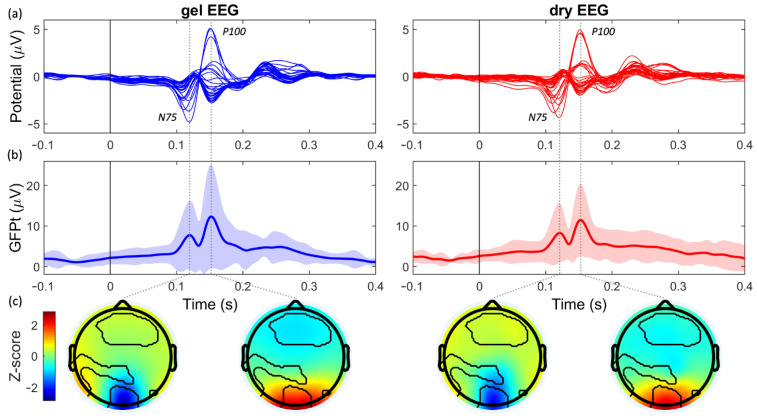
Pattern-reversal visual evoked potential (VEP) evoked by the checkerboard paradigm, for the gel EEG system (blue; left) and dry EEG system (red; right). (**a**) The average VEP (μV) per channel over subjects. (**b**) The global field power over time (GFPt; μV) averaged over subjects. Shaded areas indicate the confidence interval (mean ± 2x standard deviation). (**c**) The standardized topographic distribution (Z-score) of the two main components of the VEP. Black lines encircle the pixels that were significantly different between the two systems.

**Figure 3:**
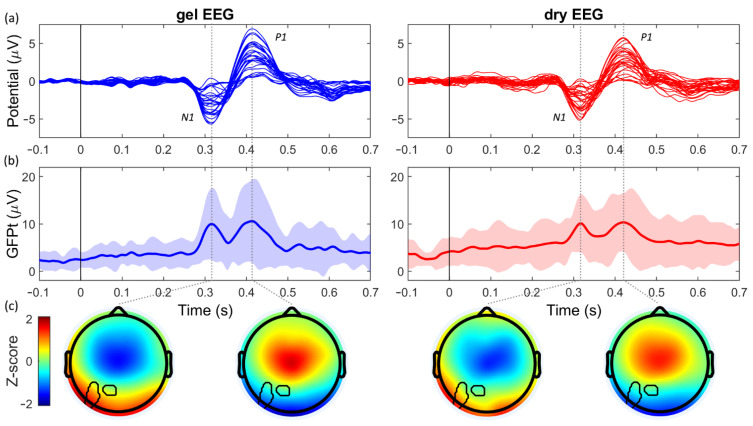
Auditory evoked potential (VEP) evoked by the auditory steady-state response task for the gel EEG system (blue; left) and dry EEG system (red; right). (**a**) The average AEP (μV) per channel over subjects. (**b**) The global field power over time (GFPt; μV) averaged over subjects. Shaded areas indicate the confidence interval (mean ± 2x standard deviation). (**c**) The standardized topographic distribution (Z-score) of the two main components of the AEP. Black lines encircle the pixels that were significantly different between the two systems.

The time signals of the evoked responses, the VEP (Figure 2a) and AEP (Figure 3a), recorded by the dry EEG system, showed strong similarities to those recorded with the gel EEG system. Moreover, the two peaks resulting from the first and second main components could clearly be observed in the GFPt of the VEP (N75 and P100; Figure 2b) and AEP (N1 and P1; Figure 3b). The average amplitudes and the latencies of these components of the GFPt were highly comparable between both systems (Table 1). Moreover, very strong within-subject correlations and small RMSD values were observed between the two systems for the GFPt of the VEP (ρ = 0.89 ± 0.10; RMSD = 1.33 μV ± 0.77 μV; median ± SD) and the AEP (ρ = 0.81 ± 0.13; RMSD = 2.27 μV ± 1.03 μV; median ± SD). 

### 3.2. Performance in the Frequency Domain

**Figure 4:**
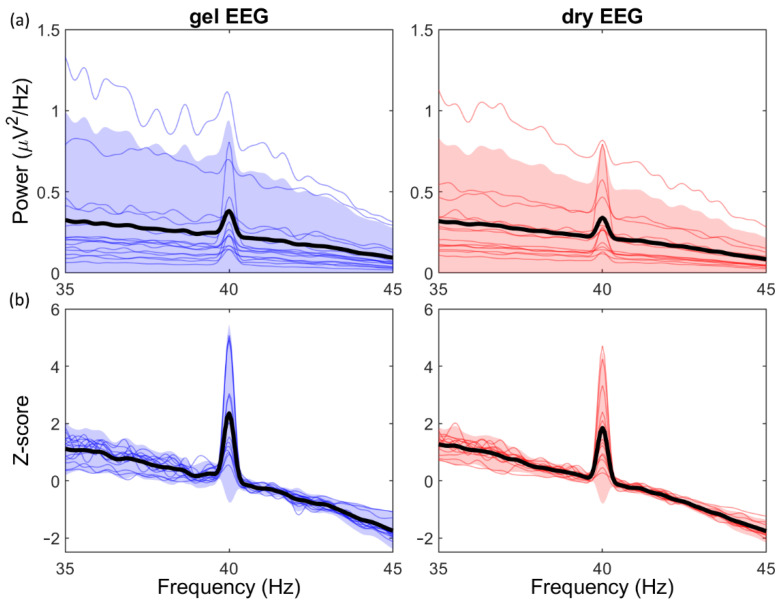
Power spectral density (PSD) of the 40 Hz Auditory steady-state response (ASSR) for the gel EEG system (blue; left) and dry EEG system (red; right). (**a**) Absolute PSD of the 40 Hz-ASSR (μV^2^/Hz), averaged over channels per subject. (**b**) Standardized PSD of the 40 Hz-ASSR (Z-score), averaged over channels per subject. Black lines indicate average PSD over all subjects and channels; shaded areas indicate the confidence interval (mean ± 2xSD).

The ASSR-task resulted in a clear 40 Hz peak in the recordings of both systems, which can be observed in both the absolute PSD (Figure 4a) and standardized PSD (Figure 4b). The absolute 40Hz-ASSR power was not significantly different between the two systems (*p* = 0.25; dry: 0.34 μV ± 0.23 μV; gel: 0.38 μV ± 0.28 μV; mean ± SD). However, the standardized 40 Hz-ASSR power was significantly decreased for the dry EEG system (*p* < 0.05; dry: Z = 1.84 ± 1.33; gel: Z = 2.35 ± 1.56; mean ± SD). The Spearman’s rank correlation coefficient of the 40 Hz-ASSR power, calculated over all pairs of electrodes within-subjects, was moderate for both the absolute PSD (ρ = 0.66, *p* < 0.001) and standardized PSD (ρ = 0.72, *p* < 0.001).

The SNR was significantly decreased for the dry EEG system for the absolute PSD (*p* < 0.01; dry: 5.50 ± 3.30; gel: 6.97 ± 4.75; mean ± SD), as well as for the standardized PSD (*p* < 0.01; dry: 2.57 ± 3.12; gel: 3.83 ± 4.57; mean ± SD). However, the Spearman’s rank correlation coefficient was again moderate for the absolute data (ρ = 0.71, *p* < 0.001) and the standardized data (ρ = 0.72, *p* < 0.001).

**Figure 5:**
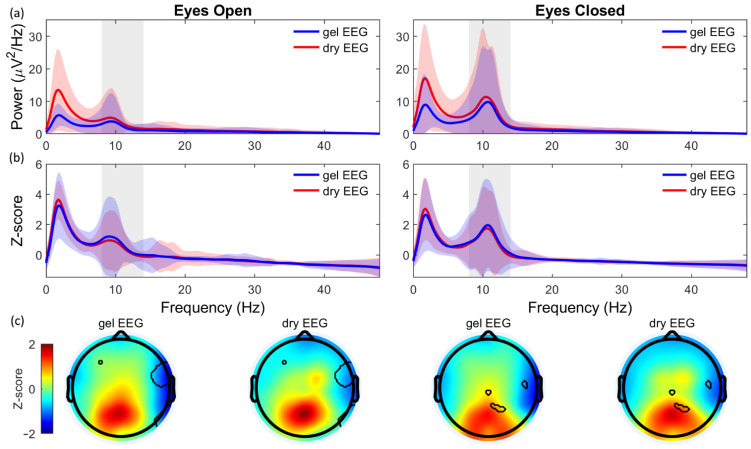
Power spectral density (PSD) of the eyes open/eyes closed task for the gel EEG system (blue) and the dry EEG system (red). (**a**) Absolute PSD (μV^2^/Hz) during the period of open eyes (left) and a period of closed eyes (right). (**b**) Standardized PSD (Z-score) during the period of open eyes (left) and a period of closed eyes (right). Lines indicate the average power over all channels and subjects. Shaded areas indicate the confidence interval (mean ± 2xSD). (**c**) The standardized topographic distribution (Z-score) of the alpha band during eyes open (left) and eyes closed (right) for the gel EEG system and the dry EEG system. Black lines in the topographic distribution encircle the pixels that were significantly different between systems.

Both the absolute (Figure 5a) and standardized PSD (Figure 5b) of the EOEC-task clearly showed a peak in the alpha band during the EC condition, while this peak was less pronounced during the EO condition. The absolute power was significantly increased for the dry EEG system in the delta and theta band during EO and EC and in the alpha band during EO (Table 2). The increase in absolute power for the dry EEG system was most pronounced for the delta band, as supported by a poor and fair correlation during both EO (ρ = 0.27, *p* < 0.001) and EC (ρ = 0.37, *p* < 0.001), respectively. However, the absolute power of the alpha band showed a moderate correlation during EO (ρ = 0.69, *p* < 0.001), and a very strong correlation during EC (ρ = 0.86, *p* < 0.001).

For the standardized PSD, the power was significantly decreased for the dry EEG system in the alpha band during EO only (Table 2). In addition, Spearman’s rank correlation showed high similarities between the two systems in the standardized PSD. With the exception of the gamma band during EO (ρ = 0.52, *p* < 0.001), moderate correlations were found for all frequency bands during both EO and EC, with a strong correlation for the alpha band (ρ = 0.81, *p* < 0.001) during EC.

### 3.3. Performance in the Spatial Domain

The P100 component of the VEP, respectively, 34.8% and 20.1% of the pixels were significantly different between the two systems (Figure 2c). For the N1 and P1 components of the AEP, respectively, only 4.1% and 0.0% of the pixels were significantly different between the systems (Figure 3c).

Moreover, the topographic distribution of the alpha band was highly comparable between both systems (Figure 5c). For EC, only 1.2% of the pixels were significantly different between systems. For the EO, 5.9% of the pixels were significantly different.

### 3.4. General Performance

**Figure 6:**
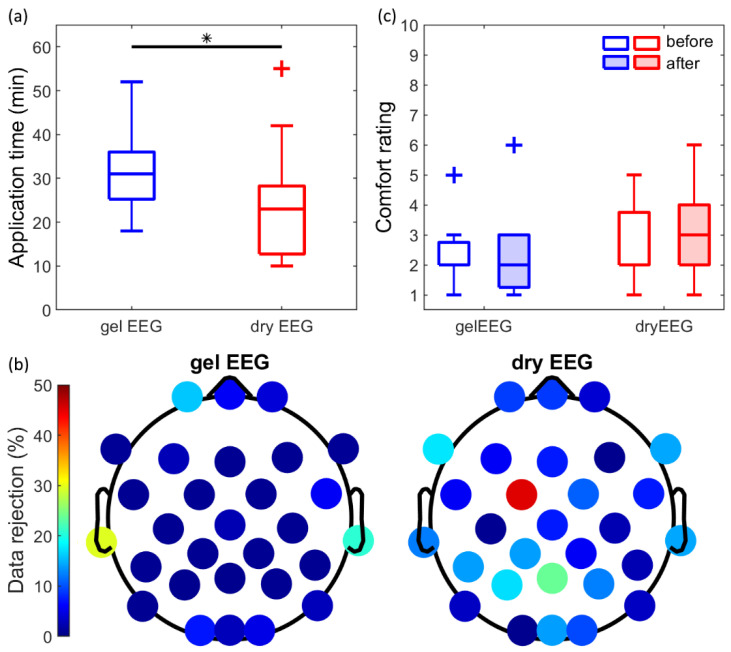
General performance. (**a**) Application time (min) for the gel EEG system (blue) and the dry EEG system (red). (**b**) Percentage of data rejection (%) per channel averaged over subjects for the gel EEG system (left) and the dry EEG system (right). 0% = no data rejected; 100% = all data rejected. Color bar from 0–50%. (**c**) Experienced comfort for the gel EEG system (blue) and the dry EEG system (red), rated by the subject before (open) and after the experiment (shaded). 1 = extremely comfortable; 10 = maximum imaginable pain. * *p* < 0.05.

The application time was significantly reduced by 8 min (*p* < 0.05; Figure 6a) for the dry EEG system (23 ± 13 min; median ± SD) in comparison to the gel EEG system (31 ± 10 min; median ± 2xSD). However, the percentage of rejected data was significantly increased for the dry EEG system (*p* < 0.05; Figure 6b). For the dry EEG system, 10.5% ± 5.7% (mean ± SD) of the data was rejected per subject, while for the gel EEG system, 3.9% ± 2.7% (mean ± SD) of the data was rejected per subject.

Moreover, there were no significant differences in experienced comfort between the two systems (Figure 6c) when taking time (i.e., the comfort rating before or after the experiment) into account (χ^2^ (1) = 3.075, *p* = 0.08). The experienced comfort before the experiment was rated as 2.0 ± 1.2 and 2.0 ± 1.0 (median ± SD), and after the experiment as 3.0 ± 1.5 and 2.0 ± 1.3 (median ± SD), for the dry and gel EEG system, respectively. The level of sleepiness of the participants did not differ between the experimental sessions with either the dry EEG system or the gel EEG system (χ^2^ (1) = 2.020, *p* = 0.155).

## 4. Discussion

The current study validates the soft, multipin, dry EEG electrode system by comparing its signal quality in the time, frequency, and spatial domains and its general performance with the signal quality and performance of a conventional gel EEG electrode system. The findings indicate similar signal quality in the time, frequency, and spatial domains for the two systems. The soft, dry electrodes measured electrical brain activity with similar accuracy as compared to conventional gel electrodes while maintaining good comfort. Therefore, the dry EEG system, consisting of soft, multipin dry electrodes, is a promising technique for use in future out-of-laboratory experiments.

### 4.1. Performance in the Time Domain

The time signals of the evoked responses, the VEP and AEP, recorded by the dry EEG system showed strong similarities to those recorded with the gel EEG system. Both the general evolution over time, assessed by the Spearman’s rank correlation (ρ > 0.8), and the absolute amplitudes of the VEP and AEP, assessed by the RMSD (RMSD < 2.3 μV), reveal the good performance of the dry EEG system. These results are in accordance with the previous results of Fiedler et al. [11], who evaluated comparable PU-based multipin dry electrodes with Ag/AgCl coating. They reported an average correlation of 0.8 and an average RMSD of 2.9 μV for the VEP between a dry and gel EEG system. These findings show that the dry EEG system can be used for future investigations on event-related potentials with similar accuracy as compared to a conventional gel EEG system.

### 4.2. Performance in the Frequency Domain

The absolute and standardized PSD of both the dry EEG system and the gel EEG system showed a clear 40 Hz peak induced by the ASSR-task and a clear increase in alpha band activity in response to a period of closed eyes. Standardization is a frequently used method that converts absolute values into a standard score (i.e., Z-score) in order to make a better comparison between channels or between subjects. Therefore, in the current study, both the absolute and standardized PSD were calculated.

The 40 Hz-ASSR power was not significantly different between systems for the absolute PSD, while the standardized 40 Hz-ASSR power was slightly decreased for the dry EEG system. A previous study of Grummett et al. [39] found a similar decrease in the power of the absolute 16 Hz and 23 Hz visual steady-state response for gold-coated multipin dry electrodes in comparison to standard gel electrodes. In addition to the 40 Hz-ASSR power, the ASSR-task was used to compare the SNR of the two systems. Consistent with the previous studies evaluating gold-plated dry electrodes [17,18], a slightly decreased SNR was found for the dry EEG system in both the absolute and standardized PSD. However, Spearman’s rank correlation over all possible pairs of electrodes showed moderate correlations for the 40 Hz-ASSR power and SNR in both the absolute and standardized PSD.

The absolute and standardized PSD of both the dry EEG system and the gel EEG system showed a clear increase in the alpha band during a period of closed eyes in comparison to a period of open eyes. High similarities could be observed between the two systems for both the absolute and standardized PSD. However, the absolute power of the delta band was significantly increased for the dry EEG system. This noticeable increase in the delta band for dry electrodes is frequently reported [10,12,14,18,20,39] and might be explained by a less stable skin-electrode contact due to the lack of conductive gel. Therefore, the dry electrodes might be more susceptible to movement artifacts or to changes in hydration of the skin (e.g., sweating), resulting in a low-frequency drift. However, the current results show a strong correlation for the frequency band of interest, i.e., the alpha band, especially during eyes closed (absolute PSD: ρ = 0.86; standardized PSD: ρ = 0.81). This strong correlation for the alpha band during an EOEC-task is reported in the literature as well [10,13,18,20].

In summary, the signals measured with the dry EEG system showed a distinct 40 Hz-ASSR power and a clear increase in the alpha band during EOEC, with slightly increased noise levels. Moreover, the signals of the dry EEG system strongly correlated with the signals of the gel EEG system. Therefore, the dry EEG system can be used properly in future investigations involving similar tasks.

### 4.3. Performance in the Spatial Domain

The topographical distributions of the main ERP components were comparable for the two systems. With the exception of the N75 component of the VEP, the pixels that were significantly different between the two systems were located primarily outside the main regions of interest of the VEP (i.e., the occipital area), and the AEP (i.e., the frontal/central area), similar to previous findings evaluating comparable PU-based multipin electrodes with Ag/AgCl coating [11]. It is remarkable that the percentage of significantly different pixels is increased for the VEP components in comparison to the AEP components. This can be explained by the faster evolution of the VEP over time and the slower and more smooth evolution of the AEP over time. Therefore, the fixed time points set to calculate the topographical distribution might include more variability for the VEP in comparison to the AEP. Another explanation is the low electrode density in the occipital area in comparison to the more densely covered frontal/central area. Larger interpolation areas for the VEP might have induced a higher number of significantly different pixels between the two systems.

Additionally, the topographical distribution of the alpha band was similar for the two systems, with less than 6% of the pixels being significantly different. The significant pixels were mainly located in the frontal-temporal region, outside the main region of interest (i.e., the parietal/occipital area). The significant differences in the topographical distribution might be caused by the interpolation of the frontal-temporal region because of the low density of electrodes and the increased percentage of rejected data within this region.

The number of significantly different pixels for the VEP and alpha band power is slightly increased in comparison to a previous study of Fiedler et al. [11], evaluating comparable multipin dry electrodes. This can be explained by the different spatial resolution due to a lower number of electrodes in the current study (32-channels vs. 97-channels), thereby producing larger interpolation areas and increased impact of channel dropouts.

### 4.4. General Performance

In the current study, the general performance of the dry EEG system was evaluated by the application time, the experienced comfort, and the percentage of rejected data. The application time of the dry EEG system was significantly decreased by 8 min, which is a reduction of 25%. One extreme outlier could be observed for the dry EEG system (Figure 2), with an application time of 55 min. This was caused by technical problems related to the hardware on which the recording software was installed, resulting in a delay of approximately 15 to 20 min. The application time is frequently reported as the main advantage of dry EEG systems. Previous studies evaluating comparable PU-based multipin dry electrodes with an Ag/AgCl coating observed a reduction from 33% (64-channel gel vs. 64-channel dry EEG system [12]) up to 86% (128-channel gel vs. 97-channel dry EEG system [11]) in application time when correcting for the number of electrodes. However, others reported an increased application time for the dry EEG system (64-channel gel vs. 33-channel dry EEG system consisting of (2 pins) spring-loaded sliver electrodes [13]). A confounding factor responsible for the high variability in results might be the prior experience of the experimenter with the application of one of the caps, as discussed by Kam et al. [13]. Therefore, it is worth noting that the experimenter of the current study had no previous experience in the application of either dry or gel EEG systems. Another possible explanation might be the differences in the number of electrodes of the systems used in studies [10]. During the application of a gel EEG system, every single electrode is handled individually to prepare the gel and obtain a good signal quality. In contrast, only the channels without a direct good signal quality are handled individually for the dry EEG system. Therefore, it can be expected that the difference in application time between the two systems is more pronounced with an increasing number of electrodes.

The experienced comfort was not significantly different between the dry and gel EEG system when taking time (i.e., the rating before and after the experiment) into account. As reported by Di Fronso et al. [12], the experienced comfort might be improved further if the experiment involves the subject performing a specific task, which distracts from the sensation of the EEG cap.

Despite the promising general performance regarding application time and comfort, the dry EEG system performed slightly worse compared to the gel EEG system with respect to the percentage of rejected data, consistent with previous studies [11,12,13,14]. This might be caused by a less stable electrode-skin contact, either due to the softness of the electrodes or due to the lack of conductive gel. It is important to point out the remarkably high percentage of data rejection for the FC1 electrode in the dry EEG system. A possible explanation might be the insufficient adduction and skin contact of the electrode. Nevertheless, only 10.5% of the data per subject was rejected for the dry EEG system, compared to 3.9% for the gel EEG system, which results in a difference of 6.6%. It must be expected that the percentage of rejected data for the dry EEG electrodes influenced the SNR. A decrease of the SNR for both the dry and gel EEG systems must be expected if the analyses were performed on the entire dataset (including the respectively rejected data during pre-processing). This aspect highlights bad channel and artifact detection and rejection, which are well established for conventional gel EEG systems [40,41,42,43,44], to be even more important for dry EEG systems [42,43]. It is important to note that our results provided enough data to compare the two systems, including statistical analyses.

In the current study, a threshold (mean + 2xSD) was used to eliminate noisy channels or epochs. Even though this method might have missed some of the noisy epochs in either the dry or gel EEG system, it is more objective than visual inspection of the signals by raters as the same criteria were used for both systems.

### 4.5. Limitations and Recommendations

For the dry EEG system, external self-adhesive drop leads were used as the reference and ground electrodes and were positioned at the mastoids. It is important to note that a tiny drop of conductive gel was applied on the drop leads to improve the impedance. Moreover, only the impedance of the ground and reference electrodes were considered for the dry EEG system. The recording software (eego^TM^ software v1.9.1, ANT Neuro b.v., Hengelo, The Netherlands) provides a ‘Dry-EEG module’, in which the signal quality, instead of the impedance, can be used to judge the raw signals. Therefore, the current study did not compare the electrode impedances between the two systems [11,12,14].

In the current study, the signal quality was determined by the software based on the raw data, and the raw data was visualized and exported. Although applicability and performance depend on the specific field of application and environmental conditions, algorithms for the automatic detection and correction of artifactual channels [40,41,42,43,44] can be beneficial for measurements outside the laboratory performed by less trained experimenters, or if the subject applies the cap themselves during experiments at home. The dry EEG system, in combination with such algorithms, should ease and speed up the preparation of the EEG system even more, making it applicable for experiments at home.

The experimental protocol included three different tasks to evaluate the performance and signal quality of the dry EEG system. The checkerboard task and the EOEC-task were used in previous validation studies, whereas the ASSR-task has not been used before. Not all subjects showed a prominent 40 Hz peak power, just as not all subjects showed a clear increase in the alpha band activity during a period of eyes closure because of inter-subject variability. However, the ASSR-task is a valuable addition to experimental protocol because of the several advantages. First, the ASSR-task can be used to evaluate multiple aspects of signal quality. The AEP can be used to compare the signal quality in the time and spatial domains, while the 40 Hz-ASSR power can be evaluated in the frequency domain. Additionally, the SNR can be determined quite well because it is explicitly known what the ‘signal’ (i.e., 40 Hz-ASSR power) should be. Second, the ASSR is a good addition to the frequently used EOEC-task, as it creates the possibility to evaluate the signal quality within multiple frequency bands. Whereas the analysis of the EOEC-task mainly focuses on the lower frequency bands, specifically the alpha band, the ASSR-task can be used to compare the signal quality of systems in the higher gamma frequency band. Moreover, the EOEC-task is used to compare frequency bands, while the ASSR-task can be used to evaluate the sensitivity of the systems to detect the power of one specific peak frequency. Third, the ASSR might be a suitable task for the validation of EEG systems during gait or cycling to evaluate susceptibility to motion artifacts. Although the available literature is limited, Yokota et al. [45] effectively used the ASSR to assess workload during walking. Taken together, the ASSR-task can be a valuable addition to the study protocol of future EEG validation studies.

A limitation of the study is that the VEP and AEP were delayed. In the current study, a delay of approximately 50 ms for the VEP and 100 ms for the AEP could be observed. These latencies are likely caused by a systematic problem in the experimental script regarding the presentation of visual and auditory stimuli or the transfer of event-markers between systems. Since it is a systematic delay, similar for all subjects and experimental sessions, it affected the recordings with both electrode systems equally and therefore did not invalidate the further analysis and interpretation of the data in case of the comparative validation study at hand.

The current study strongly suggests that the dry EEG system can be used in future studies for the measurement of brain activity in stationary tasks. However, further research should be performed to investigate the signal quality of the soft dry electrodes during mobile tasks, such as walking or cycling, to evaluate the effect of motion artifacts. Moreover, it would be interesting to observe the effect of an increased measurement time on the signal quality and the experienced comfort for both systems.

## 5. Conclusions

The current study showed that the soft, multipin, dry EEG electrode system could be used for future investigations and studies on electrical brain activity during stationary tasks, with similar accuracy as compared to a conventional gel EEG system. The signal quality in the time, frequency, and spatial domains were comparable for the two systems, with slightly increased noise levels for the dry EEG system. Moreover, the general performance of the system was promising, with a reduced application time and only a slight increase in the percentage of rejected data. Therefore, it can be concluded that the dry EEG system is a promising technique for future out-of-laboratory applications requiring rapid and/or intermittent measurements.

## Figures and Tables

**Table 1:** The amplitudes (μV; mean ± SD) and latencies (s; mean ± SD) of the main components of the visual evoked potential (N75, P100) and the auditory evoked potential (N1, P1), for the dry and the gel EEG system.

	Dry EEG		Gel EEG	
	Amplitude (μV)	Latency (s)	Amplitude (μV)	Latency (s)
*Visual evoked potential*				
N75	8.64 ± 3.61	0.121 ± 0.005	8.26 ± 4.41	0.120 ± 0.006
P100	11.81 ± 4.39	0.153 ± 0.004	12.66 ± 6.42	0.152 ± 0.003
*Auditory evoked potential*				
N1	10.99 ± 3.07	0.313 ± 0.020	11.13 ± 3.61	0.320 ± 0.014
P1	12.05 ± 3.96	0.410 ± 0.030	12.15 ± 3.80	0.402 ± 0.027

**Table 2:** Descriptive statistics (mean ± SD) of the absolute (μV^2^/Hz) and standardized (Z-score) power spectral density (PSD) per frequency band during the eyes open/eyes closed (EOEC)-task for the dry EEG system and gel EEG system. *p*-value (*p*-Val) after Bonferroni correction as a result of the Wilcoxon signed-rank test and Spearman’s correlation coefficient (rho) are indicated for each frequency band and condition.

	Absolute PSD				Standardized PSD			
	Dry EEG	Gel EEG	*p*-Val	Rho (ρ)	Dry EEG	Gel EEG	*p*-Val	Rho (ρ)
*Eyes open*								
Delta	10.28 ± 4.73	4.51 ± 1.29	**	0.27 **	2.62 ± 0.51	2.37 ± 0.80	0.010	0.76 **
Theta	4.54 ± 2.20	2.61 ± 1.37	**	0.45 **	0.82 ± 0.23	0.88 ± 0.37	0.208	0.66 **
Alpha	3.27 ± 1.86	2.40 ± 1.75	*	0.69 **	0.48 ± 0.41	0.64 ± 0.48	*	0.79 **
Beta	1.14 ± 0.79	0.80 ± 0.65	0.022	0.34 **	−0.24 ± 0.24	−0.23 ± 0.20	0.208	0.71 **
Gamma	0.52 ± 0.51	0.38 ± 0.40	0.056	0.41 **	−0.53 ± 0.06	−0.55 ± 0.05	0.083	0.52 **
*Eyes closed*								
Delta	12.66 ± 5.89	6.64 ± 2.78	*	0.37 **	2.09 ± 0.73	1.83 ± 0.83	0.041	0.78 **
Theta	5.71 ± 3.08	3.66 ± 1.90	*	0.52 **	0.63 ± 0.28	0.67 ± 0.38	0.678	0.70 **
Alpha	7.39 ± 4.87	6.08 ± 4.23	0.013	0.86 **	0.96 ± 0.55	1.10 ± 0.57	0.013	0.81 **
Beta	1.26 ± 0.72	0.92 ± 0.62	0.018	0.54 **	−0.33 ± 0.10	−0.31 ± 0.11	0.151	0.70 **
Gamma	0.51 ± 0.42	0.34 ± 0.36	0.073	0.48 **	−0.52 ± 0.06	−0.53 ± 0.06	0.390	0.68 **

* *p* < 0.01 (α-level after Bonferroni correction); ** *p* < 0.001.

## Data Availability

Pre-processed data, processed data, and analysis scripts are available from the Data Sharing Collection of the Donders Repository via the following URL: https://doi.org/10.34973/ytxa-qn07. Raw, unprocessed data are stored in the local Data Acquisition Collection and Research Documentation Collection of the Donders Repository and are available upon request to the corresponding author (j.j.a.heijs@utwente.nl).
